# Can you believe what you read in the papers?

**DOI:** 10.1186/1745-6215-10-55

**Published:** 2009-07-16

**Authors:** Mike Clarke

**Affiliations:** 1 School of Nursing and Midwifery, Trinity College Dublin, 24 D'Olier Street, Dublin 2, Ireland

## Abstract

The number of reports of clinical trials grows by hundreds every week. However, this does not mean that people making decisions about healthcare are finding it easier to obtain reliable knowledge for these decisions. Some of the information is unreliable. Systematic reviews are helping to resolve this by bringing together the research on a topic, appraising and summarising it. But the quality of these reviews depends greatly on the quality of the studies, and this usually means the quality of their reports. If there are fundamental flaws within a study, such as the use of inappropriate 'randomisation' techniques in the context of reviews of the effects of interventions, the reviewers will not be able to fix these. Worse still, if they are not aware of underlying flaws, they might make incorrect judgements about the quality of the research in their review. A study by Wu and colleagues of 'randomised trials' from China provides a reminder of the cautious approach needed by users of scientific articles. They contacted the authors of more than 2000 research articles, which purported to be reports of randomised trials; and concluded that ten of every 11 studies claiming to be a randomised trial probably did not use random allocation. Better education of researchers, peer reviewers and editors about what is, and is not, a properly randomised trial is needed; along with better reporting of the details for how participants were allocated to the different interventions. Systematic reviewers must be cautious in making assumptions about the conduct of trials based on simple phrases about the trial methodology, rather than a full description of the methods actually used. It's not that you can't believe anything that you read in the papers, just that you cannot believe everything.

## Introduction

There are ever increasing numbers of papers available in healthcare journals, and even more articles appearing in newer media such as the Internet. At first sight, the depth and breadth of this material might mean that people making decisions about their own care or that of others have never had it so good. Surely, they will be able to find research in the relevant topic area. They will. But the problem is: some of this research might not be reliable and the decision maker might not be able to find a sufficiently unbiased collection of the research to help her to make the right decision.

## Discussion

For decisions about the effects of health care, randomised trials should boost the chances that comparisons are not confounded by factors other than the interventions being compared. They are, therefore, a more reliable guide for estimates of the differences between the actual interventions [1]. However, the problem of publication bias means that trials which have findings that do not favour the experimental intervention are less likely to be published quickly or at all [2,3], making the available literature a potentially biased and unreliable source of knowledge.

It is possible that the recent growth in the number of trial reports being published is a sign that publication bias is being overcome. During this first decade of the twenty-first century, at least 25,000 reports of randomised or controlled trials have been published each year [4]. However, we will not know if this is a fair reflection of the volume of research being done until recent initiatives on widespread trial registration provide a means of tracking large cohorts of trials over time, and there may still be some way to go before all trials are registered prospectively. For example, the World Health Organisation's International Clinical Trials Registry Platform shows that nearly 20,000 trials were registered in the constituent registers in 2008, an increase of more than 4000 compared to 2007 [4]. However, it will only be through the routine reporting of the findings of all trials that publication bias will be eliminated [5].

In the last two decades, the growth in another type of research article might also hold out some hope for decisions makers. Systematic reviews bring together the findings of the individual studies relevant to a specific question, and thousands are published each year [6]. But, the authors of these reviews face the challenge that the quality of their work depends greatly on the quality of the studies they review, which usually means the quality of the reports of these studies. If there are fundamental flaws in the underlying study design, such as the use of inappropriate 'randomisation' techniques, the researchers working on the systematic review will not be able to fix these. Worse still, if they are not aware of the underlying flaws, they might make incorrect judgements about the quality of the research they are reviewing.

This brings us to the study by Wu and colleagues of 'randomised trials' in what may become one of the largest sources of healthcare research evidence, China [7], and its reminder that readers of scientific articles need to be cautious in their interpretation. Wu *et al*. gathered together more than 3000 research articles that had been published in Chinese journals in just over 10 years from 1994, which purported to be reports of randomised trials. They conducted telephone interviews with the authors of 2235 of these reports and found that their answers were indicative of the conduct of a properly randomised trial for just 207 of these reports. Ten of every 11 reports claiming to be a randomised trial probably were not.

This work confirmed what had been found by some of the authors on a smaller scale within the context of systematic review of Chinese medicinal herbs for treating measles. Of the 28 reports of randomised trials they found, Gu *et al*. were able to contact the authors of 19. Their discussions led them to conclude that none of these were properly randomised trials, casting sufficient doubt on the other nine studies for the authors to decide that none of the studies should be included in their review [8].

Wu *et al*. suggest a few reasons for the poor quality of the information reported for this large amount of Chinese research, and point to some solutions. These will need to include better education of researchers, peer reviewers and editors about what is, and is not, a properly randomised trial. Better reporting of how the participants in a trial were allocated to the different interventions will also be needed to help the reader to decide for herself whether or not a trial being described as 'randomised', really was; as proposed in the CONSORT statement [9].

## Conclusion

The message for systematic reviewers is to continue to be cautious in making assumptions about the conduct of trials based on simple phrases about the methodology of the research, rather than a full description of the methods actually used. Decision makers in health care need to be presented with reliable evidence in order to make their decisions as reliable as possible. People conducting prospective studies need, therefore, to ensure that the information they present is reliable and systematic reviews need to appraise this information carefully and to distinguish between the label on a study and what really went on "inside the tin".

It's not that you can't believe anything that you read in the papers, just that you cannot believe everything.

## Competing interests

My salaried employment relates to the conduct of systematic reviews and randomised trials. Therefore, I have an interest in ensuring that the importance of this type of research is recognised by people making decisions about healthcare and research funding. I also have an interest in ensuring that systematic reviews are done reliably, in order to maximise their benefits to the health and wellbeing of individuals and populations.
